# Wings on concealed corpse: the forensic importance of scuttle flies (Diptera: Phoridae)

**DOI:** 10.3389/finsc.2025.1727384

**Published:** 2026-01-19

**Authors:** Yali Guo, Yifei Luo, Yuting Ma, Afito Luciano, Jingjing Huang, Ye Li, Shiwen Wang, Yuequn Niu, Zhou Li, Jifeng Cai, Fanming Meng

**Affiliations:** 1 Department of Forensic Medicine, School of Basic Medical Sciences, Xinjiang Medical University, Urumqi, Xinjiang, China; 2 Department of Parasitology, Central South University, Changsha, China; 3 Shaoyang Center for Disease Control and Prevention, Shaoyang, Hunan, China; 4 Key Laboratory of Forensic Medicine, Xinjiang Medical University, Urumqi, China; 5 Department of Forensic Science, School of Basic Medical Sciences, Central South University, Changsha, Hunan, China

**Keywords:** corpses, forensic entomology, insect, phorid fly, postmortem interval

## Abstract

Forensic entomology focuses on insects associated with decomposing remains to provide clues for forensic investigations. Among those insects, scuttle flies are uniquely capable of colonizing enclosed spaces and underground burial sites where other insects cannot access, often serving as the sole entomological “witnesses” to provide crucial evidence in forensic practice. This review highlights the forensic significance of scuttle flies, including the biological characters, diversity and behaviors of scuttle flies from forensic aspects based on reported cases. We investigate the biological and environmental factors influencing their utility in forensic investigation, and scuttle fly species commonly found on corpses were also summarized. Additionally, suggestions on future research directions of scuttle flies and how their biological characteristics can enhance their application in forensic entomology are also provided.

## 1 Introduction

Forensic entomology is a branch of applied entomology that uses the presence of necrophagous insects to assist in criminal investigations, such as by estimating the minimum post-mortem interval (PMI_min_), inferring changes in the corpse’s position, and so forth, to provide auxiliary evidence (1). In forensic research, the accurate assessment of the time of death, the postmortem interval (2), occupies an extremely critical position (3). Flies act as important forensic indicators as they are the foremost visitors of the corpse and act as silent witnesses after the accomplishment of a crime (2).

Forensically relevant insects (such as scuttle flies, flesh flies, and house flies) can rapidly locate and colonize a freshly deceased body. However, when the corpse is situated in a relatively enclosed environment (such as sealed containers, locked rooms, buried coffins) (4–6), a physical barrier is formed by theses conditions, which hinders their colonization. Based on this, two key forensic questions arise. First, the delay in egg-laying time (from several hours to several days or weeks) may lead to an underestimation of the PMI. Because traditional indicators based on insect development time do not take these barriers into account (7). Although isolated reports describe Phoridae insect associated with long-buried or skeletonized remains (8), such occurrences represent exceptional and highly specific conditions rather than typical Diptera colonization patterns. Diptera generally cannot oviposit or complete development on fully skeletonized bodies, where beetles dominate the fauna (9, 10). Second, these enclosed environments stabilize the decomposition process by reducing temperature fluctuations, maintaining constant humidity, reducing air flow, and weakening competition among insect species (11), which further modifies colonization dynamics and development rate of insects.

Scuttle flies are among the most species-rich and worldwide distributed groups of insects (12). Adult scuttle flies are small, ranging from 0.75~8.0 mm in size, and the larvae are 1~4 mm. Adults exhibit colors such as dark black, gray-brown, or yellowish. Adults have an enlarged thorax with a characteristic humpbacked appearance. Adults and larvae prefer moist environments and live on decaying carrion, carcass, dung, plants, flowers, fungi, beehives, or anthills (13).

Due to the smaller body size and the natural ability to locate food sources in a relatively confined environment, scuttle flies tend to be found in crime scenes that other insects cannot reach, which gives them special meaning during forensic investigation (14). Lutz et al. (15) found that 949 insect-related cases, 80% entomological evidence, can provide day-specific PMI_min_ estimates. **Table 1** summarizes the scuttle flies found on human corpses in forensic cases and their estimation of PMI_min_.

**Table 1:** Scuttle flies found on human corpses in forensic cases and their estimation of PMI.

Scuttle flies found on human corpses in forensic cases and their estimation of PMI					
Species	Distribution area	Cadaver type	Scene	PMI	Reference
*Megaselia scalaris* Loew	Buenos Aires, Argentina	skeletonized	Soft wooden coffin under 50 cm deep in the grave	4-8y	Mariani et al. (5)
	Chiang Mai, Thailand	Mummified and partial skeletonied	Under a tree in the forest and near the road	4.5mos	Sukontason et al. (16)
	Guangdong, China	skeletonized	Soft-shell zipper suitcase	56d	Hu et al. (17)
	Bonn, Germany	Body mummified, face skeletonized	Windows and door closed	37d	Reibe and Madea (18)
		Advanced decay	All windows closed	33d	
		Advanced decay	All windows closed	15d	
	Central Italy	Active decay	Windows and doors closed	21d	Bugelli et al. (19)
		Initial active decay	Windows partially closed	2d	
		Advance decay, skeletonized	Windows and doors closed	25-43d	
	Kuala Lumpur, Malaysia	Advanced decay	Only a window open	8-9d	Syamsa et al. (20)
			A kitchen window open	10-14d	
			Bedroom and kitchen windows open	9-13d	
	Penang, Malaysia	Mummified	Fully closed house	13d	Thevan et al. (21)
		Active decay	7th -floor apartment	3-5d	
*Megaselia curtineura* Brues					
*Megaselia spiracularis* Schmitz					
		Fresh		1d	
	Bari, Southern Italy	Mummified	A dry cellar 15 meters underground	20m	Introna et al. (4)
	Guangdong, China	Advanced decay	Windows partially closed	11-12d	Li et al. (22)
	Selangorr, Malaysia	Mummified	Indoor	7d	Ivorra et al. (23)
*Megaselia rufipes* Meigen	East of England	–	A cupboard, the door sealed with “Mastik” type sealant and the handle removed	100d	Disney and Manlove (24)
*Megaselia abdita* Schmitz		–	A shallow grave	77d	
		–	A deep 0.5m grave in a woodland	53-63d	
		Dried out	Indoor	18w	Manlove and Disney (25)
*Triphleba nudipalpis* Becker		–	Grave	25d	Disney and Manlove (26)
*Conicera similis* Haliday	Valencia, Spain	Skelotonized	Close, watery and dark tank with minimum ventilation	>1y	García-Rojo et al. (27)
*Dohrniphora cornuta* Bigot					
*Dohrniphora* sp	Buenos Aires, Argentina	Skelotonized	Soft wooden coffin -40 cm deep in the grave	4-8y	Mariani et al. (5)
*Conicera tibialis* Schmitz	Battle Creek, Michigan	Wreckage stage	Embalmed corpose with a depth of 1.8 m in an unsealed casket	28y	Merritt et al. (28)
*Puliciphora rufipes* Silva Figueroa	Alicante, Spain	Active decay, slightly mummified except the head	Under an open beach umbrella, wrapped in a blanket up to the neck	30d	Ivorra et al. (29)

Considering the unique behaviors and potential value in forensic investigation, this review aims to: 1) expound the forensic significance of scuttle flies under specific environments in cases, 2) analyze the factors influencing scuttle flies' colonization of decomposing remains, and 3) identify the species of scuttle flies of forensic significance. mos=month, y=year, d=day

## 2 The forensic application scenarios of Scuttle flies

### 2.1 Buried corpses

Phoridae species are among the most typically encountered taxa on decaying carcasses in the soil at various depths (30). Many cases have been reported of Phoridae being found on bodies in graves, and most of the bodies were found skeletonized (5, 24, 26). In a case, the phorid fly is the only insect found on the corpse of a murder victim buried (<1 m depth) in a heavy clay soil in the East of England (26). It was reported to colonize carcasses at a depth of 60 centimeters in less than two weeks post-burial (31). *Megaselia scalaris* Loew could enter through the smallest holes to reach the buried carcasses, due to its tiny body size (32). *M. scalaris* can also detect carrion buried in coffins at a depth of around 1.83metres and oviposit on it, providing a food source for the larvae that hatch from the eggs, supporting their development until pupation (33).

The rate of decomposition of buried bodies is highly dependent on the depth of the burial and the ambient temperature (34). In an extreme case, *Conicera tibialis* Schmitz can invade corpses buried for years and successfully complete at least one generation of reproduction, where a saponified corpse was buried in a zinc coffin at a depth of about 2 meters (8). Although exceptional cases exist, Phoridae generally cannot breed on fully skeletonized remains. Saponification of corpses can slow down the degree of corruption (35). Since it can provide sufficient physical resources for Phoridae and support multi-generation reproduction, their presence in buried bodies is considered part of the cadaveric fauna and is not a reliable indicator for postmortem interval estimation. In addition, insect invasion of the buried remains will also impact the decomposition rate and pattern (36). It should be considered when estimating the postmortem interval.

### 2.2 Wrapping corpses and closed space

Firstly, to make the evidence disappear, some suspects would place the corpse in a relatively closed environment such as a quilt, a vehicle, or a suitcase (17, 37, 38). Due to the influence of the containing environment, wrapping will prolong the process by which insects search for corpses and reduce the number of eggs laid. In this regard, different wrapping methods will cause differences in the time when flies first come into contact with the corpse. For instance, plastic bags, simulated luggage, and plastic bags plus suitcases will gradually prolong the time for flies to come into contact with corpses for the first time (39). This is mainly attributed to differences in material, thickness, and breathability. While wrapping caused a 1 to 13 days delay in the arrival of other species, Phoridae did not exhibit any delay in reaching the carcass (40). In addition, differences in the exposure level of the corpse, its location, as well as other aspects of the discovery scene, will further influence the colonization process of the flies by altering the ease with which they come into contact with the corpse (39, 41).This phenomenon is consistent with the research of Andrews et al. (7), Phoridae were most abundant in the luggage, as opposed to the wheeled bins and the exposed environment. Cases that further support this view include: Ivorra et al. (29) found *Puliciphora. rufipes* Silva Figueroa on a body wrapped in a blanket. Similarly, Zuha et al. (42) reported that *Puliciphora* Dahl (Diptera: Phoridae) was found on rabbit carcasses placed in luggage and garbage. Besides, they also recorded that *Dahliphora sigmoides* Schmitz, *Gymnoptera simplex* Brues, *M. scalaris*, *Puliciphora borinquenensis* Wheeler, *Puliciphora obtecta* Meijere, and *Spiniphora* sp. were found on rabbit carcasses confined to sealed plastic bins (43). Due to the dual interference of insect colonization time and decomposition rate, this eventually leads to an impact on the accuracy of PMI_min_ inference for wrapped corpses.

In addition to insect colonization, the wrapping also significantly alters the process of corpse decomposition. The decomposition time of package processing is longer than that of unpackage processing (44). This is due to plastic bags isolating the samples from the external environment, creating anaerobic conditions that promote fat decomposition and the formation of adipocere (35). Simultaneously, the impermeability of plastics leads to the accumulation of decomposition products during the degradation process, which slows down the decomposition rate (45). When comparing the corpses wrapped in plastic with those in an open environment, it was found that the duration of the fresh stage was the same for both, but the duration of the wrapped corpses in the remaining stages was longer (46). Because plastic wrapping creates a high-humidity and low-oxygen environment, which inhibits the activity of putrefactive bacteria and slows down tissue decomposition (47, 48). But it creates a high temperature environment and increases insect activity (49).

However, the influence of the characteristics of the wrapping material itself on decomposition is more complex. When comparing cotton fabric with plastic, cotton fabric causes local dryness due to its hygroscopic properties, delaying decomposition. It is worth noting that mulch made of natural fibers has been proven to accelerate the formation of liposis compared with that made of synthetic fibers in aquatic environments. The reason for this phenomenon is that natural fibers have a stronger water absorption capacity than synthetic fibers (50). This difference may stem from absorption. It may remove the decomposition products of the surface tissues of the corpse. This effect may disrupt the decomposition process and make the formation of fat more likely to occur.

Lastly, the interaction between the package and other environmental factors also needs to be considered. During the interaction between soil burial and wrapping, it was observed that the proportion of mass loss (the ratio of post-burial to pre-burial quality) indicated wrapping had a greater impact on the decomposition of corpses, but no impact on TBS (51).

### 2.3 Indoor building

Scuttle flies (Diptera: Phoridae), particularly *M.scalaris*, hold unique forensic significance in indoor death scene investigations. Unlike dominant necrophagous species like *Lucilia sericata* Meigen and *Calliphora vicina* Robineau-Desvoidy (Calliphoridae), Phoridae exhibit remarkable adaptability to enclosed environments (19, 52). Their tiny body size enables them to colonize cadavers in sealed indoor spaces through narrow gaps in doors or windows, even when these openings are only partially closed (18). This ecological trait explains their frequent dominance in indoor insect communities despite their relatively low abundance in regional surveys. For instance, while Phoridae accounted for merely 0.39% of the insect fauna in human cadavers across Tehran District (53), they were successfully documented in an indoor case within the same region, highlighting their niche specialization.

The vertical distribution patterns of scuttle flies further enhance their forensic utility. Although primarily associated with ground-level indoor cases (20), *M. scalaris* demonstrates exceptional resource exploitation capabilities in high-rise environments (6). Field experiments reveal they can colonize substrates at heights exceeding 100m through passive transport mechanisms and human-mediated dispersal (54), outperforming Calliphoridae and Sarcophagidae in vertical colonization efficiency (55). This behavioral adaptation positions Phoridae as critical PMI_min_ indicators in high-rise death scenes where conventional blowfly evidence is absent. While current case reports linking Phoridae to elevated crime scenes remain limited (20), their predictable colonization patterns in confined spaces warrant increased attention from forensic entomologists. However, Phoridae are not strictly dependent on human cadavers; they can also develop in other substrates such as garbage, sewage pipes, and decaying plant materials. Therefore, samples collected from substrates associated with cadavers are applicable for PMI estimation.

### 2.4 Myiasis

Myiasis is an infestation of living humans or animals with dipterous larvae that feed on the host’s live or dead tissue, liquid matter, or ingested food from their host, and cause a wide range of infections depending on body location and the larvae’s relationship to the host (56). Necrophilic insect species found on human and animal corpses are also present in patients with myiasis. (57). Once the host dies and the tissues are fully decomposed, the same fly species may behave as necrophagous colonizers rather than as myiasis agents. Therefore, in forensic investigations it is important to distinguish true antemortem myiasis from postmortem colonization of cadavers by necrophagous larvae. *M. rufipes*, *M. scalaris*, and *Megaselia* sp*iracularis* Schmitz were seen in cases of myiasis (58–60). Myiasis tends to show up in neglected cases. Under these conditions, the possibility of antenatal myiasis infection needs to be carefully considered when evaluating cases and PMI_min_ (61). Diptera larvae can infest open wounds, predisposing the patient to severe secondary bacterial infection and, if untreated, even sepsis and death, which may indicate serious neglect or abuse (62). Critically, myiasis associated flies like *M. scalaris* exhibit ecological plasticity: they infest both living hosts and cadavers, necessitating caution when linking larvae solely to PMI_min_ calculations. Standardized protocols should integrate wound microbiology, larval species identification, and developmental data to avoid misinterpreting antemortem infestations as postmortem colonization events.

## 3 Factors affecting scuttle flies on corpses

### 3.1 Temperature

The growth rate of insects varies with temperature. Studying the development rate of insects at different temperatures is conducive to predicting more accurate PMI_min_ (63). Wang et al. (64) found that *M. scalaris* development duration decreased with increasing temperature in the range of 16-34°C. Thomas et al. (65) explored the growth rate of *M. scalaris* at 24°C, 28°Cand 32°C, respectively, increased by 32.1%, 13.9% and 45.5%, which indicates suitable temperatures promoting quicker development. Zuha and Omar (66) asuggested that *M. scalaris* larval length is possibly not ideal indicator for estimating larval age at temperatures less than 28°C and greater than 33°C. Feng et al. (67) found that the mean time of the pupation stage was negatively correlated with temperature for *Dohrniphora cornuta* Bigot. At lower temperatures and wetter conditions, adult lifespan increased for Phoridae (68).

Some species of Phoridae exhibit low temperature tolerance, and their adults can be active on snow surfaces at temperatures as low as -4°C (69, 70). Phoridae are active in the cold season, and most blowflies are inactive due to the low temperatures (25). Seasons can affect the distribution of phoridae indoors. Almutawa et al. (6) reported that adult phorids are only found indoors in winter. However, it was not found either indoors or outdoors in summer. While Phoridae maintain activity in winter indoor environments (18.0-19.6°C), elevated summer temperatures (38.1-43.0°C) may restrict their survival and colonization potential. Low temperature can cause developmental arrest. *Megaselia giraudii* Egger undergoes diapause during the pupal stage, with the emergence time extended by 184–187 days compared to normal conditions (71). Although current research has not directly confirmed the complete developmental history of Phoridae in winter or low-temperature environments. Therefore, the PMI_min_ estimation, based on a comprehensive consideration of this biological characteristic, may have significant reference value when determining death cases in the cold season.

### 3.2 Humidity

Phoridae exhibit the highest abundance during the early decomposition stage (72). As an indicator species for this stage, this characteristic can serve as an auxiliary reference for inferring PMI_min_. During the fresh and bloated stages, the moisture content of the natural cadaveric openings might be as high as 70% to 80% (73), creating an ideal microenvironment for their activity. Under relatively humid conditions (> 75% RH), the lifespan of Phoridae will be prolonged (68). Experimental studies on *M. scalaris* demonstrate that substrate moisture significantly impacts pupal recovery and adult emergence. At 50% humidity, larvae complete development rapidly, whereas at 90% humidity, survival remains unchanged, but developmental time extends by approximately two days, and adult emergence rates drop by 20.64% (74). This is the reason why it is difficult for them to colonize over a long period of time. Furthermore, human cadaver moisture fluctuates across decomposition stages, directly affecting Phoridae colonization patterns. For forensic applications, this underscores the necessity of incorporating cadaver water content into PMI_min_ estimates. Thus, when estimating PMI_min_ for bodies with varying moisture levels, careful calibration based on humidity-driven developmental thresholds is essential to ensure reliability.

### 3.3 Burial depth and soil

Post-burial interval estimation, similar to PMI_min_, focuses on the development of data from insects detected on buried carcasses. The timing and pattern of insect colonization on buried corpses are determined by soil type and depth (75). Scuttle flies may arrive at corpses buried in deep soil in earlier phases of decomposition and may develop huge populations (76). Pastula and Merritt (31) reported that *M. scalaris* colonized corpses at a depth of 60 cm in the soil and was collected 7 days after burial, which can help estimate Post-burial interval (PBI) for forensic investigation. Moreover, it was discovered in excavated bodies buried in wooden coffins at a depth of 30–60 cm in the soil. AL-Mekhlafi et al. (77) found larvae of *M. scalaris* under 20 cm at the decay and advanced decay stage of decomposition on buried rabbits. *Triphleba nudipalpis* Becker appeared on the body of a murder victim buried in a meter of clay (26). *Conicera similis* Haliday was collected from a rat carcass buried 40 cm depth (78). *C. tibialis* can be found on corpses that have been buried at a depth of 2m for 18 years (8). Besides, soil moisture affects the growth and development of *M. scalaris*, the larval stage lasts the longest, and the pupation rate is the lowest in the soil with 0% water content (79). Therefore, when dealing with burial cases, it is also necessary to consider the impact of soil moisture on the growth and development of insects.

### 3.4 Drugs intake, pollutants, and toxic substances

Commonly, overdose-related deaths happen in isolated regions, particularly among lonely victims, frequently discovered in the latter stages of decomposition (80). Insects can be used as substitute evidence in poisoning death investigations (81). By analyzing larvae that feed on corpses, it is possible to identify the drugs present in decaying corpses (82). Insects colonize charred corpses, and poisons taken before death can be detected in the larvae (83).

Miller et al. (84) reported that *M. scalaris* pupae were discovered in the mummified body of a female drug addict who died 33 months earlier in her home. Several added drugs were found in the body. The pupae were found to contain amitriptyline and nortriptyline in the following analysis. The drug inhibits larval growth in a concentration-dependent manner (85). Clonazepam could expedite the growth of larvae, reaching their pupal stage earlier (86). *M. scalaris* that gnaw on carcasses in the presence of malathion experience reduced egg and pupa viability, diminished larval growth, and an extended larval stage, which can lead to errors in PMI_min_ inference (87). In addition, a particular drug or poison can have different effects on different species (88). The drug’s effect on larval development might alter the agreement between the entomological PMI_min_ estimation and the circumstantial facts in this situation. Current research indicates that there is no linear relationship between morphine concentrations in necrophagous insects and their larval feeding periods, as morphine does not undergo bioaccumulation within the insects (89). The efficacy of toxicological detection depends on whether the absorption rate of the drug in the host organisms exceeds its metabolic clearance rate. This dynamic equilibrium limits existing technologies to qualitative analyses of toxins in insect samples (e.g., confirming the presence or absence of specific substances) rather than enabling quantitative correlations with the postmortem interval (PMI_min_). Therefore, while toxicant detection holds significant referential value in forensic science, its sole application remains insufficient to support accurate estimation of the PMI_min_.

Because of the faster growth rate, the larval age may be overestimated. Alcaine-Colet et al. (90) reported that *M. scalaris* could complete its life cycle when fed on modeling clay, a substrate containing toxic inorganic compounds, which results in increased larval development time, reduced pupal development time, and a shorter adult life duration of *M. scalaris*. The effects of pollutants on the development of *M. scalaris*, such as hexavalent chromium and four species of selenium (91–93). Exploring insect toxicology can therefore estimate the minimum postmortem interval (94) and the circumstances of death in which toxic substances are suspected to have caused the death (88).

Interestingly, there are some reports of gasoline homicide. Gasoline can delay the time when scuttle flies enter the body. Rumiza et al. (95) used gasoline to mimic homicide or suicide. It has been established that the existence of gasoline and its odor on the corpse delays the decomposition process by postponing the entry of insects by 6 hours.

### 3.5 Nocturnal oviposition

Estimation of time has passed since death corresponds to the PMI_min_, which is based on the age of the oldest insects detected on bodies. Commonly, necrophagous flies like blowflies or flesh flies are inactive at night; hence, oviposition happens during the day (96). However, *M. scalaris* can lay eggs in complete darkness both during the day and at night, and the number of eggs laid at night is very similar to that laid under continuous dark conditions (97). If the insects in the discussion were nocturnal and laid eggs at night, it might cause the PMI_min_ estimation to be off by up to 12 hours. Unlike Calliphoridae, common and representative research subjects in forensic entomology, which typically oviposit under sufficient daylight with extremely low egg production at night (98). *M. scalaris* can access the oviposition location and lay eggs in complete darkness both during the day and night. Nevertheless, the quantity of eggs laid during the day is larger. Moreover, the number of eggs laid during the night under LD conditions (12 hours of light, followed by 12 hours of darkness) is remarkably close to the amount in complete darkness (97). This implies that in situations where there are frequent periods of darkness, the number of eggs deposited is enough to support the population. It is advised that nocturnal oviposition should be included when using scuttle fly as a reference for PMI_min_ estimation. Further, efforts to correlate numerous elements that contribute to nocturnal oviposition, such as temperature, relative humidity, and bait type, should be given.

### 3.6 Other factors

Dimorphism is an essential factor distinguishing between male and female development stages. The size dimorphism of *M. scalaris* is obvious during post-feeding and pupa stages, such as female post-feeding larvae and puparia are larger than males, and females have a longer total developmental period than males at almost all temperature ranges (66). It could help investigators estimate the development time of the species more accurately, thereby correctly estimating the development time of the species. Thus, the dimorphic difference should be considered when interpreting developmental data for PMI_min_ analysis.

## 4 Scuttle flies of forensic interest

Phoridae is among the most biologically diverse and species-rich groups of insects, which contains 250 genera and about 3,400 species worldwide (12, 99). **Table 1** summarizes the scuttle flies found on human corpses in forensic cases in terms of Phoridae species, distribution area, cadaver type, scene, and PMI_min_ inference time.

### 4.1 Megaselia

*M*. *scalaris* is the most studied forensic-related scuttle fly for estimating the post-mortem interval of human remains (100). It is widely distributed and can be found in various habitats, including urban structures and tropical rainforest (101). It is a polyphagous species and can live on various foods, including decomposing organic materials, artificial media, and decaying substrate (102, 103). Additionally, *M. scalaris* is commonly associated with indoor death or neglect cases of humans or household animals (104).

This species played a significant role in decaying bodies located indoors or in concealed environments due to its small size and ability to penetrate these regions earlier than other insects (18, 21, 105). It is the only insect discovered breeding within a tightly closed seventh-floor flat in Japan (106). It was found on exposed pig carrion after 5–12 days of exposure (107). It is a common and typical forensic indicator that was found in the decay, advanced decay, and dry stages (32). Zhang et al. (108) found that the mean developmental duration of *M. scalaris* from egg to adult stage needs 417.7 hours at 25°C and an intrinsic optimum temperature of 21°C.

In a Phoridae study in Egypt, *Megaselia curtineura* Brues was the most abundant species, except *M*. *scalaris* (109). It is also recorded among the 16 species associated with 50 human remains in the tropical climate of northern Malaysia (110). Thevan et al. (21) first reported the use of the growth and development of *M. curtineura* for PMI_min_ inference in forensic cases.

*M.* sp*iracularis* had the longest development time (1131.1 h) at 16°C and the shortest development time (232.6 h) at 34°C (64). Although *M.* sp*iracularis* and *M. scalaris* are closely related, their total developmental history at room temperature differs by 50.7 hours (64).

### 4.2 Dohrniphora

*D. cornuta* is a common species of Phoridae fly found indoors in China, and has also been reported in England, Spain, and Sweden, indicating that it is a widespread species (111, 112). Adults collected in a house in the Pennines (U.K.) at 380 meters above sea level were most likely attracted by a neighboring septic tank (113). The species was also collected on skeletonized human remains inside a closed tank (27). In addition, it developed faster at 24°C and 30°C, but was restricted beyond 36°C (67).

### 4.3 Conicera

*C. tibialis*, the coffin fly, is distinctive among the species commonly observed on buried bodies due to its peculiar association with cadavers in coffins and limited spaces. As a typical species adapted to the burial environment, *C. tibialis* can penetrate soil up to 2 meters deep to lay eggs and form multiple generations of reproduction in closed coffins (114). Martín-Vega et al. (8) further discovered that it could complete the life cycle on a corpse buried for 18 years. This species was discovered in a concrete coffin buried 1.8 m deep, 28 years after death (28). This breakthrough record not only reveals the ecological resilience of this species under extreme time spans but also challenges the boundaries of the traditional PMI_min_ estimation model in forensic entomology.

*C. similis* has been found on both skeletonized human remains and decayed rat corpses, and these findings collectively confirm its ability to colonize dead bodies (27, 78).

### 4.4 Triphleba

*T. nudipalpis* is one of the most representative species. This species is found in the British Isles, continental Europe from Sweden to France, and Russia’s far east from the Czech Republic to Hungary (26). Larvae of *T. nudipalpis* feed on buried carcasses and are well-known soil breeders, as indicated by the large amount of phenology data acquired with emergence traps placed over the soil (115). Adults of *T. nudipalpis* have been reported visiting carrion (26). The larvae have been subsequently shown to be feeders on buried carcasses (116). Furthermore, they have been proven to be efficient in locating buried carrion, as is proven by the fact that pieces of flesh were buried at varying depths, and flies were reported as early as four days after burial (117). Conversely, many female phorids visit carrion for a protein-rich feed, which assists in the growth of their eggs, rather than laying mature eggs (118).

### 4.5 Puliciphora

Species of this genus are distributed worldwide and found primarily in warm-climate zones (119, 120). *P. borinquenensis* is a species of forensic importance in buried or closed environments (121). *P. borinquenensis*, *P. obtecta*, and *Puliciphora beckeri* Meijere were discovered on rabbit carcasses, as well as in luggage and trash cans (42). *Puliciphora rufipes* Silva Figueroa was found wrapped in a blanket (29).

## 5 Conclusion and perspectives

Compared to other insect species of forensic importance, scuttle flies have specific significance in forensic science, especially in cases involving bodies in enclosed spaces or environments. The tiny body size and necrophagous feeding habit may partly explain their advantage under such conditions. Traditional carbiophilic flies (such as those from the Calliphoridae family) can locate corpses because their antennae contain chemoreceptors and they are relatively sensitive to cadaverine and putrescine. Although the tiny size of the Phoridae is regarded as its main advantage in penetrating closed environments, the special sensitivity of its chemoreceptors may be underestimated. This mechanism has not yet been systematically verified in forensic practice. However, the biological mechanisms of why and how scuttle flies locate and invade corpses in enclosed spaces are not yet fully understood. In the future, it needs to be further explored in combination with molecular biology techniques (such as olfactory receptor gene analysis).

In enclosed spaces, the developmental patterns and oviposition behavior of scuttle flies are crucial for estimating PMI_min_ (122). Previous research has collected data on the life stages of Phoridae, but much of this information originates from controlled laboratory settings with stable temperatures. However, this does not adequately reflect the complex environmental conditions encountered in real-world scenarios. Compared to open environments, factors such as temperature, humidity, and food availability within confined spaces are more stable, which affects the body decomposition and insect development in a unique manner. Moreover, the oviposition behavior of scuttle flies is also influenced by environmental conditions. Therefore, further exploration of the developmental patterns and oviposition behavior of Phoridae in simulated enclosed spaces is essential for improving the accuracy of PMI_min_ estimation. In certain criminal scenarios, such as deep burial and chemical treatment of corpses, they may be the only reliable evidence. Looking to the future, it is necessary to promote the transition of Phoridae from “alternative tools” to “core tools”. This requires in-depth cooperation between the forensic medical community, entomologists, and data scientists to build a globally shared multi-omics database of Phoridae.

In recent years, advanced technological methods have been applied in forensic entomology field. To fully realize the potential of Phoridae as forensic indicators, future research must adopt advanced technological approaches currently available in forensic entomology. Key future directions include the application of multi-omics techniques to decipher their unique biological characteristics in concealed environments. Currently, machine learning algorithms have been used to model the developmental data of other necrophagous insects and cadaver microbiome succession independently for PMI_min_ estimation (123–125). However, the integrated analysis of these two datasets remains a missing link. Constructing models that represent the entire “cadaver-Phoridae-microbiome” system represents a crucial objective for achieving the next key breakthrough in accurate PMI_min_ estimation.

Scuttle flies are crucial in forensic entomology, especially in enclosed environments where other insects cannot access. Their small size and adaptability allow colonization of buried, wrapped, or indoor corpses, serving as unique “witnesses” for PMI_min_ estimation. Factors like temperature, humidity, burial depth, and toxins influence their development and colonization, with species like *M.scalaris* demonstrating nocturnal oviposition and drug tolerance. Forensic cases worldwide highlight their role in extreme scenarios. Future research should explore molecular mechanisms of their environmental adaptation and build global multi-omics databases to enhance PMI_min_ accuracy in concealed death scenes.
